# Fluorescence imaging for a noninvasive *in vivo* toxicity-test using a transgenic silkworm expressing green fluorescent protein

**DOI:** 10.1038/srep11180

**Published:** 2015-06-10

**Authors:** Yoshinori Inagaki, Yasuhiko Matsumoto, Masaki Ishii, Keiro Uchino, Hideki Sezutsu, Kazuhisa Sekimizu

**Affiliations:** 1 Laboratory of Microbiology, Graduate School of Pharmaceutical Sciences, The University of Tokyo, 7-3-1 Hongo, Bunkyo-ku, Tokyo 111-0033, Japan; 2 Transgenic Silkworm Research Unit, National Institute of Agrobiological Sciences, 1-2 Owashi, Tsukuba, Ibaraki, 305-8634 Japan

## Abstract

In drug development, the toxicity of candidate chemicals must be carefully examined in an animal model. Here we developed a live imaging technique using silkworms for a noninvasive toxicity test applicable for drug screening. Injection of carbon tetrachloride, a tissue-injuring chemical, into transgenic silkworms expressing green fluorescent protein (GFP) induced leakage of GFP from the tissues into the hemolymph. The leakage of GFP was suppressed by pre-administration of either cimetidine, a cytochrome P450 inhibitor, or N-acetyl cysteine, a free-radical scavenger. The transgenic silkworm was made transparent by feeding a diet containing chemicals that inhibit uric acid deposition in the epithelial cells. In the transparent silkworms, GFP fluorescence in the fat body could be observed from outside the body. Injection of salicylic acid or iron sulfate, tissue-injuring chemicals, into the transparent silkworms decreased the fluorescence intensity of the GFP in the fat body. These findings suggest that the transparent GFP-expressing silkworm model is useful for evaluating the toxicity of chemicals that induce tissue injury.

In the process of drug development, the toxicity of candidate compounds must be carefully evaluated. Although toxicity testing is generally performed using mammals, such as mice and rats, the required use of a large number of animals is problematic in terms of the high cost and animal welfare ethical issues. These problems have become restrictive factors for drug development^1^. Toxicity testing using cultured cells has been proposed as an alternative method to using mammals^2^, but the findings obtained using cultured cells deviate from those obtained using animals^3^, possibly due to differences in the pharmacokinetics of the chemicals in living bodies and cell cultures. As the pharmacokinetics of chemicals are influenced by factors such as administration, distribution, metabolism, and excretion^4^, screening must be performed in living animals.

To avoid the high cost and ethical issues of mammalian models, the development of effective invertebrate models is desired. *Drosophila melanogaster* and *Caenorhabditis elegans* are commonly used as invertebrate model animals in general biology. Injecting accurate volumes of sample for determination of the median lethal dose (LD_50_) is difficult, however, because the body sizes of these invertebrates are too small to be injected using syringes with needles. We have proposed the use of the silkworm as a model animal^5^. The silkworm is large enough for quantitative administration of a drug solution using standard syringes and needles. In addition, the median effective dose (ED_50_) of antibiotics in silkworms is very consistent with that in mammals^6^. Furthermore, chemicals are synthesized by common processes in silkworms and mammals; the reaction is first catalyzed by cytochrome P450 and subsequently with conjugation reactions^7^. The fat body is related to the drug metabolism like the liver in mammals. The gut is the main large organ of larvae related to absorption, metabolism and transportation of various substances. Those tissues have important roles in the pharmacokinetics in silkworm as well as in mammals. Moreover, the LD_50_ values of various cytotoxic compounds are very consistent between silkworms and mammals^7^. Together, these findings indicate that the basic pharmacokinetic features of chemicals are essentially similar between silkworms and mammals. Using a silkworm infection model for a second screening followed by minimum inhibitory concentration determination, we recently discovered a novel antibiotic, lysocin, which has therapeutic effects in a mouse infection model^8^. Silkworm is suggested to be a suitable and useful animal for screening drug candidates that have therapeutic effects without toxicity in mammals^8^.

We previously reported that injection of carbon tetrachloride, a chemical that induces tissue injury, led to an increase in the activity of alanine aminotransferase (ALT) in the silkworm hemolymph^9^. This finding suggests that chemical toxicity that induces tissue injury can be evaluated using silkworms, without killing the animal. Our previous protocol, however, required the invasive manipulation of harvesting the hemolymph from the silkworm, making it difficult to collect continuous data from the same individual animal. To overcome this problem, we aimed to establish a noninvasive method for evaluating the toxicity of drug candidates in silkworms.

“Visualization” is a simple and effective way to continuously evaluate adverse events in living systems. Visualization of tissue injury in animals would be useful for evaluating toxic effects of candidate medicines. We considered that the expression of green fluorescent protein (GFP) in silkworm tissues might be an effective way to observe tissue injury processes in animal bodies in a noninvasive and continuous manner. Imamura and colleagues created a transgenic GFP-expressing silkworm using the GAL4-upstream activating sequence (UAS) system^10^. In the transgenic GFP-expressing silkworm, GFP expression is induced in the whole body of silkworm because the actin promoter had been used to drive the gene. When the transgenic silkworm is exposed to excitation light, the tissue expressing the GFP is visualized by the resulting fluorescence^10^. Fluorescence of GFP is stable *in vivo* for a long period of time and has high sensitivity for detection^11^. In this paper, we describe that GFP expressed in the tissue of transgenic silkworm leaks into the hemolymph due to chemical-induced tissue injury. We also established a method of making the silkworms transparent, which allowed us to continuously monitor tissue injury in living silkworms.

## Results

### Leakage of GFP into the hemolymph in transgenic GFP-expressing silkworms after injecting tissue-injuring chemicals

**Figure 1:**
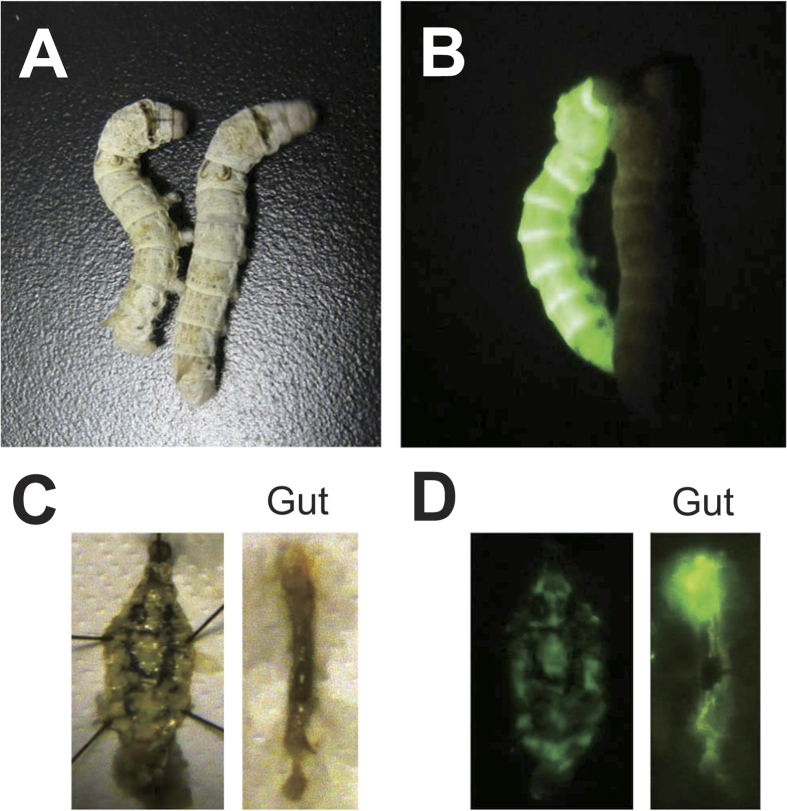
Anatomy of GFP transgenic silkworms. **A**, Bright field; **B**, Under excitation light; **C**, Dissection in brightfield; **D**, Dissection under excitation light. The left pictures of **C** and **D** show the dissected whole body after removal of the gut. The fat body was distributed under the epidermal tissue. The right pictures of C and **D** show isolated gut.

**Figure 2:**
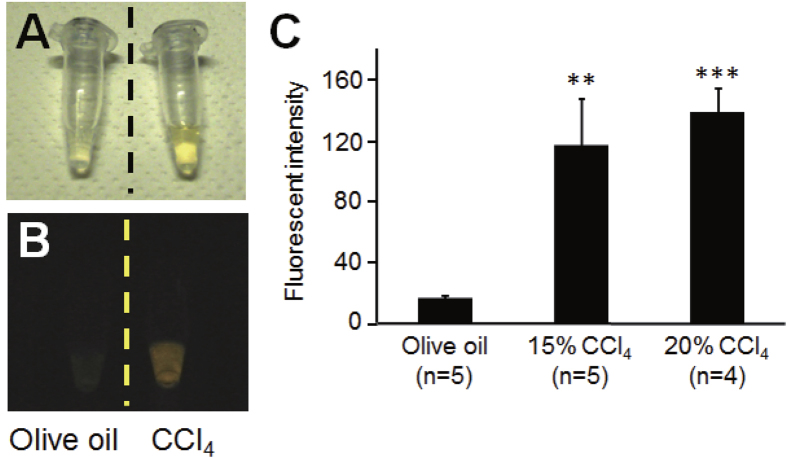
Fluorescence of hemolymph of GFP transgenic silkworms injected with CCl_4_. Silkworms fasted for 6 h were injected with 15% and 20% CCl_4_ or olive oil, and then fluorescence of the silkworm hemolymph was measured 1 day later (n = 5). **A**, In bright field; **B**, Under excitation light; **C**, Fluorescence intensity of hemolymph (mean ± SEM) **P < 0.01, ***P < 0.001.

**Figure 3:**
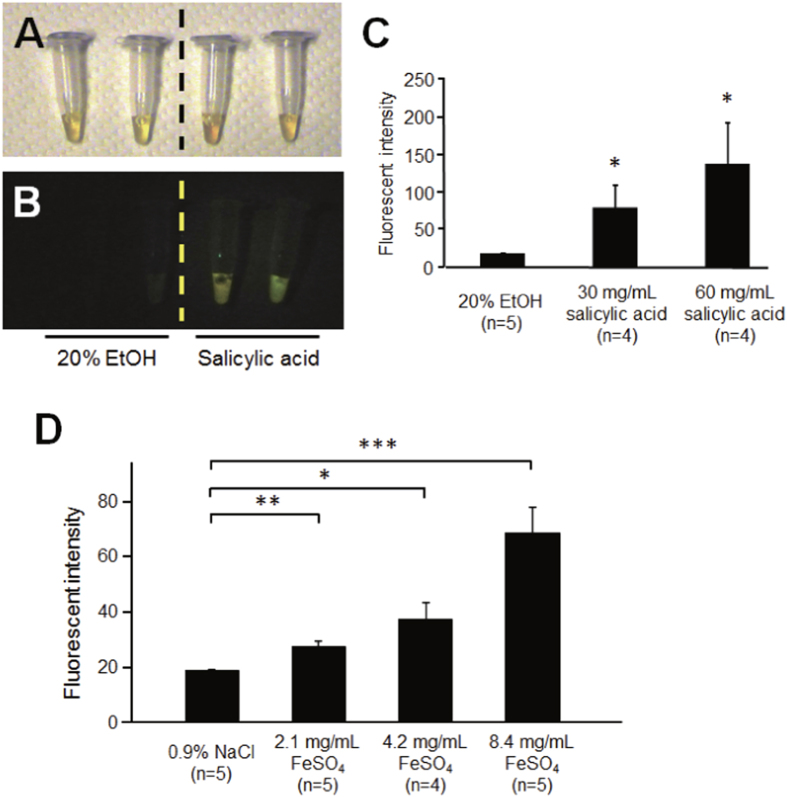
Appearance of fluorescence in hemolymph of GFP transgenic silkworms injected with salicylic acid or FeSO_4_. Silkworms fasted for 6 h were injected with various concentrations of chemicals or solvent, and reared for 1 day. Fluorescence of the silkworm hemolymph was measured (n = 5). **A**, In brightfield; **B**, Under excitation light; **C**, Fluorescence intensity of hemolymph of silkworm injected with salicylic acid (mean ± SEM); **D**, Fluorescence intensity of hemolymph of silkworm injected with FeSO_4_ (mean ± SEM). *P < 0.05, **P < 0.01, ***P < 0.001

Anatomical analysis of the transgenic GFP-expressing silkworms revealed that GFP was strongly expressed in the gut and fat body, which functions similarly to the mammalian liver (Fig. 1). Injection of carbon tetrachloride (CCl_4_) led to an increase in the fluorescence intensity of GFP in the hemolymph of the transgenic silkworm in a dose-dependent manner (Fig. 2). By contrast, no fluorescence was observed in the hemolymph of silkworms injected with olive oil, the carrier solvent for CCl_4_. This result suggests that tissue injury induced by CCl_4_ caused GFP to leak from the tissue cells into the hemolymph of the transgenic silkworm. We previously reported that injection of cytotoxic chemicals, including salicylic acid and iron sulfate, increases the activity of alanine-aminotransferase (ALT), a marker enzyme for liver injury in humans, in the silkworm hemolymph^9^. In the present study, we observed that the fluorescence intensity in the hemolymph of the transgenic silkworm increased following injection with these cytotoxic compounds in a dose-dependent manner (Fig. 3). These results suggest that tissue-injuring chemicals induced GFP to leak into the hemolymph from damaged tissues in transgenic silkworms.

### Catalytic reaction with cytochrome P450 is involved in CCl_4_-induced tissue injury in the transgenic silkworms

**Figure 4:**
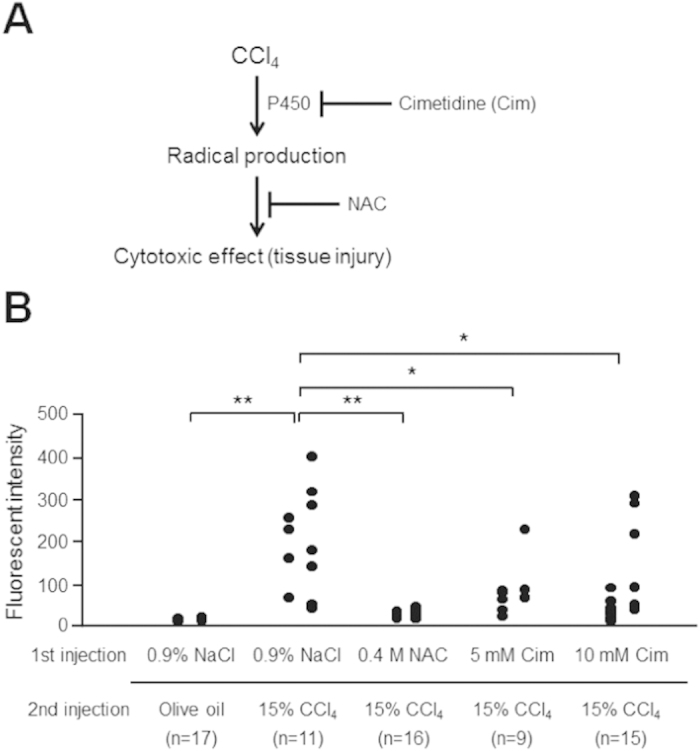
Suppression of increase in fluorescence intensity by pre-injection of NAC or cimetidine. **A**, Mechanism of tissue injury induced by CCl_4_. Working points of NAC and cimetidine are shown. **B**, Fluorescence intensity of hemolymph from silkworms injected with CCl_4_. Silkworms were fasted for 6 h, then injected with 50 μl of 0.4 M NAC, 5 mM cimetidine (Cim in figure), or 0.9% NaCl (first injection), followed 30 min later by further injection with 25 μl of 15% CCl_4_ or olive oil (second injection). After 1 day, the hemolymph was harvested to measure the fluorescence intensity. First and second line of each data means the independent assay. *P < 0.05, **P < 0.00001

In mammals, CCl_4_ generates free radicals via catalytic reaction with cytochrome P450, resulting in tissue injury^12^,^13^. We previously reported that 7-ethoxycoumarin, which is metabolized by a de-ethoxy reaction catalyzed by CYP2E1 in human liver, is also metabolized in the silkworm^7^. Here we examined whether the increase in the amount of GFP in the hemolymph of the transgenic silkworm induced by injection of CCl_4_ was due to the generation of free radicals via a catalytic reaction of cytochrome P450. The CCl_4_-induced increase in fluorescence intensity in the hemolymph of the transgenic silkworm was suppressed by pre-administration of either cimetidine, a CYP inhibitor, or N-acetyl cysteine (NAC), a free radical scavenger (Fig. 4). This result suggests that the CCl_4_-induced leakage of GFP into the hemolymph of the transgenic silkworm is due to free radicals produced by the metabolic reaction of CCl_4_ with cytochrome P450.

### A noninvasive method to evaluate the toxicity of tissue-injuring chemicals using transparent transgenic silkworms

**Figure 5:**
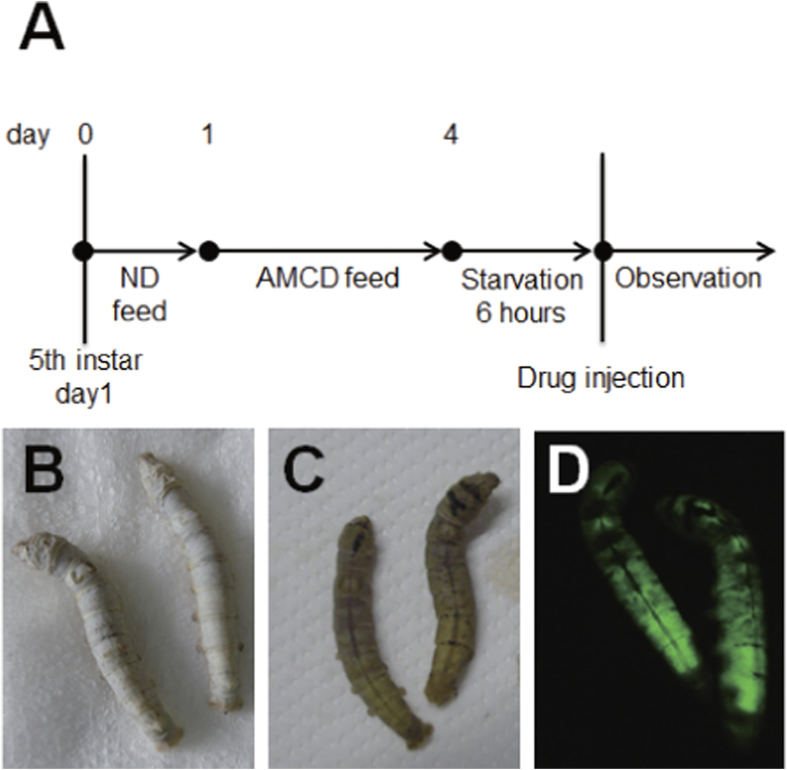
Preparation of transparent transgenic silkworms expressing GFP. **A**, Silkworm feeding protocol. Silkworms were fed a normal diet (ND) for 1 day followed by a mixed diet (AMCD) for 3 days. **B**, Normal silkworm (in brightfield); **C**, Transparent silkworm (in brightfield); **D**, Transparent silkworm (under excitation light).

**Figure 6:**
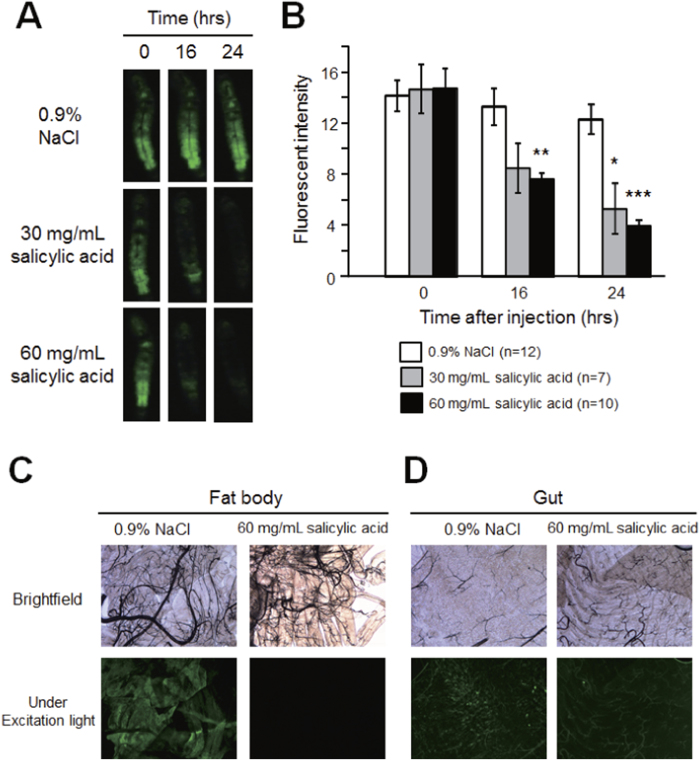
Time-dependent decrease in fluorescence intensity of transparent GFP transgenic silkworms injected with salicylic acid. Silkworms were made transparent by feeding them a mixed diet (0.5% allopurinol, 0.01% melamine and 0.5% sodium citrate). After fasting for 6 h, silkworms were injected with 0.9% NaCl or salicylic acid. Fluorescence intensity of the whole body of transparent silkworms was measured 16 and 24 h after injection. **A**, Whole-body fluorescence of transparent silkworm under excitation light; **B**, Fluorescence intensity of transparent silkworm (mean ± SEM). **C**, Fat body tissue of silkworm injected with 0.9% NaCl or salicylic acid (in brightfield and under excitation light). **D**, Gut tissue of silkworm injected with 0.9% NaCl or salicylic acid (in brightfield and under excitation light). *P < 0.05, **P < 0.01, ***P < 0.001. Original magnification of micrograph: ×5.

**Figure 7:**
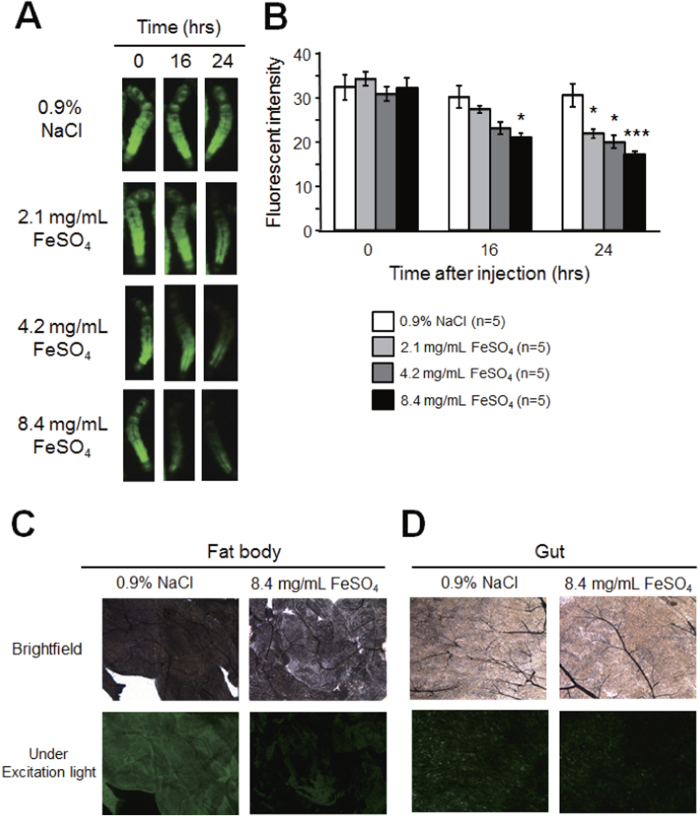
Decrease in whole-body fluorescence of transparent GFP transgenic silkworms injected with FeSO_4_. Transparent silkworms fasted for 6 h were injected with 0.9% NaCl or FeSO_4_. Whole-body fluorescence intensity was measured 16 and 24 h after injection. **A**, Whole-body fluorescence of transparent silkworm under excitation light; **B**, Fluorescence intensity of transparent silkworm (mean ± SEM). **C**, Fat body tissue of silkworm injected with 0.9% NaCl or FeSO_4_ (in brightfield and under excitation light). **D**, Gut tissue of silkworm injected with 0.9% NaCl or FeSO_4_ (in brightfield and under excitation light). *P < 0.05, ***P < 0.001. Original magnification of micrograph: ×5.

In the results described above, we harvested the hemolymph from the silkworms. The silkworm is damaged when harvesting the hemolymph, and thus it is very difficult to continuously and safely collect samples from the same animal. To solve this problem, we aimed to establish a noninvasive method by monitoring the GFP fluorescence in the silkworm, which required direct observation of the GFP fluorescence from outside the silkworm. Observation of GFP fluorescence in the tissue from outside the silkworm body is prevented by the presence of uric acid, a white pigment, on the epithelial cells (Fig. 1). A recently reported technique induces transparency of mammalian animals for improved detection of fluorescence imaging signals^14^,^15^. Tamura reported that the body surface of the silkworm becomes transparent when silkworms are fed allopurinol, a uric acid synthesis inhibitor, or melamine, an epithelial cell uric acid uptake inhibitor^16^. Citric acid also inhibits the storage of uric acid in mammals^17^,^18^. Thus, we tested the effect of feeding silkworms a diet containing allopurinol, melamine, and citric acid to induce transparency of transgenic silkworm skin (Fig. 5). As expected, the white pigment disappeared from the skin of the silkworms after feeding them the diet (Fig. 5C). Furthermore, when the transparent transgenic GFP-expressing silkworms were irradiated with excitation light, the GFP fluorescence was clearly observed at the site of fat body (Fig. 5D). We then injected the transparent silkworms with salicylic acid or iron sulfate. The GFP fluorescence intensity in the silkworms decreased following injection with salicylic acid in a time- and dose-dependent manner (Fig. 6A,B). The decrease of fluorescence intensity was observed microscopically in fat body of silkworm injected with salicylic acid (Fig. 6C). We also observed a decrease in the GFP fluorescence intensity in silkworms injected with iron sulfate in a time- and dose-dependent manner (Fig. 7A,B), and microscopically in fat body of silkworm injected with iron sulfate (Fig. 7C). The decrease of fluorescence intensity in fat body was also observed macroscopically (Fig. S1). In the gut tissue, the fluorescence intensity was low and no significant differences were observed in the silkworm injected with salicylic acid or iron sulfate compared with those injected with 0.9% NaCl (Figs. 6D,7D and S1). In assay for CCl_4_, the decrease in GFP fluorescence intensity was observed in silkworm injected olive oil as well as CCl_4_ (Fig. S2).

## Discussion

In the present study, we established a novel method for evaluating cytotoxic chemicals using a transgenic GFP-expressing silkworm. This method will be useful for performing toxicity tests for a large number of candidate compounds for drug discovery.

In mammals, CCl_4_ produces free radicals via a metabolic reaction that is catalyzed by cytochrome P450, resulting in liver injury, whereas the cytotoxic activity of salicylic acid is produced by inducing mitochondrial dysfunction and the cytotoxic effects of iron sulfate are due to the hydroxyl-radicals produced by the Fenton reaction. Here we demonstrated that injection of these tissue-injuring chemicals having different mechanisms of action into GFP-expressing transgenic silkworms led to increased fluorescence in the hemolymph. The toxic effects of these tissue-injuring chemicals may lead to tissue breakdown, causing GFP to leak from the tissues into the hemolymph of the transgenic silkworms. We previously reported a toxicity test based on measurements of ALT activity in the silkworm hemolymph^9^. The method described in this paper is much simpler than the previous method, which requires biochemical measurement of the enzyme activity.

In this paper, we demonstrated that GFP fluorescence in the tissue of the transparent GFP transgenic silkworms could be detected without killing the animal. By using this system, we could continuously observe the reduction of the fluorescence intensity in the tissue after administering the cytotoxic chemicals. The reduction of the fluorescence intensity in the tissues was due to the leakage of GFP from the injured tissue into the hemolymph. The microscopic observation showed the decrease of fluorescence intensity in fat body of silkworm injected with salicylic acid and iron sulfate. As described above, fat body and gut contain the series of enzymes, which have significant roles in metabolism of chemicals. Therefore, the injured tissues are considered to be the fat body and the gut. The GFP transgenic silkworm is established as a whole body-expressing model. However, the expression level of GFP varies among tissues. The fluorescence intensity of gut was lower than that of fat body (Fig. S1). Furthermore, the fluorescence intensity of the gut of the silkworm did not decrease by injection of salicylic acid or iron sulfate (Figs. 6D,7D and S1). Therefore, we consider that the leakage of GFP from the gut does not significantly affect the fluorescence intensity in the hemolymph in the toxicity-test. Real-time observation of chemical-induced injury in silkworms is useful for determining whether the chemical induces acute toxicity. Tissue injury is caused not only by administration of a toxic chemical, but also by infectious diseases or metabolic disorders^19^,^20^,^21^. We previously reported silkworm models of infectious disease using human pathogens, and of hyperglycemia by feeding a high glucose-containing diet^5^,^6^,^8^,^22^,^23^,^24^,^25^. Monitoring the decrease in GFP fluorescence in the fat body of the transparent silkworm might therefore be useful for observing the physiological changes caused by diseases, without killing the animal.

In drug development, candidate compounds identified by basic screening studies require further preclinical evaluation of efficacy and toxicity, which is traditionally performed in mammals^4^. A candidate compound that induces acute toxicity, such as hepatotoxicity or nephrotoxicity, does not progress to clinical trials even if it shows great efficacy in the basic studies. Preclinical testing using animals are thus very important for drug development. The use of mammals, such as mice and rats, for evaluating the toxicity of a candidate chemical is associated with high cost and ethical issues, which hinders drug development^1^. As described above, various cytotoxic compounds including CCl_4_ showed very consistent LD_50_ values between silkworms and mammals^7^,^26^. We propose the use of the silkworm as a model animal for evaluating acute drug toxicity. Silkworms are much less expensive than mammals and do not require a large space for rearing^27^. Furthermore, there is no risk of silkworms escaping the laboratory space, which is a potential biohazard. In addition, silkworms are large enough to handle, making it easy to inject them with an accurate volume of drug solution by syringe, a technique needed for quantitative measurement of therapeutic action. Moreover, silkworm body weight is approximately one-tenth that of the mouse, and therefore the amount of the testing compound needed for toxicity testing can be decreased compared to tests using mice. We consider that this method of using GFP-expressing transgenic silkworms for toxicity testing of drug candidates will overcome the cost and ethical problems associated with the use of mammals. The advantages of this silkworm testing model allow for *in vivo* toxicity testing on a large scale. Therefore, the use of silkworms for toxicity testing is expected to be an effective method for identifying candidate compounds in preclinical drug development. This testing model using transparent silkworm has a problem for the precise toxicity testing of samples dissolved in olive oil because of decreasing fluorescence by olive oil alone. Several kinds of silkworm transduced mutation in the gene related to uric acid metabolism had been known to become transparent^28^,^29^,^30^. In further study, improvement of this silkworm testing model contributes to be established a simplified model by using those transparent silkworm mutants to solve the problem of the present model.

In conclusion, we developed a noninvasive method of testing the *in vivo* toxicity of tissue-injuring chemicals by monitoring GFP fluorescence in the tissue of transparent transgenic GFP-expressing silkworms. Application of this screening method for drug discovery will contribute to reducing the number of mammals used to develop new therapeutic compounds.

## Methods

### Reagent

Carbon tetrachloride (CCl_4_), salicylic acid, cimetidine, allopurinol, and sodium citrate were purchased from Wako Pure Chemical Industries, Osaka, Japan. Iron sulfate (FeSO_4_) was purchased from Koso Chemical Co., Ltd., Tokyo, Japan. N-acetyl cysteine (NAC) was purchased from Sigma-Aldrich, St. Louis, MO. Melamine was purchased from Nacalai Tesque Inc., Kyoto, Japan.

### Transgenic silkworms expressing GFP

Transgenic GFP-expressing silkworms were established using the GAL4/UAS system described in a previous report^10^. Briefly, silkworms expressing GFP in response to the UAS promoter (UAS-GFP) were crossed with silkworms expressing GAL4 in response to the actin A3 gene promoter and DsRed in response to the promoter, which can induce compound multifaceted eye-specific expression (A3-GAL4). An A3-GAL4 line 193-2, which drives ubiquitous expression of GAL4, was used in this study^31^. Silkworms expressing both GFP and RFP in compound multifaceted eyes were screened under a fluorescence microscope and established as GFP transgenic silkworms.

### Evaluation of drug-induced tissue injury based on the fluorescence intensity of the hemolymph

Fifth-instar silkworm larvae on day 1 were fed Silkmate 2S (Nosan Corporation, Yokohama, Japan) for 1 to 2 days at 27 °C. After the body weight increased to 1.5 to 2.0 g, they were fasted for 6 h, and solution containing a cytotoxic compound was injected into the hemocoel from the backside of the larvae. Liposoluble compounds such as CCl_4_ were dissolved in olive oil, and injected (25 μL/silkworm) using a glass syringe (MICROLITER^TM^ #710, Hamilton Co., Reno, NV) with a 27G needle. Hydrosoluble compounds such as salicylic acid and iron sulfate were dissolved in 0.9% NaCl (Saline) and injected (50 μL/silkworm) using a disposable syringe (Terumo Corporation, Tokyo, Japan) with a 27G needle. After incubation at 27 °C for 1 day, the hemolymph was collected. Fifty-fold diluted hemolymph was used for measurement of the fluorescence intensity (Ex, 475 nm; Em, 509 nm) with a fluorometer (FP-6200; JASCO International Co., Ltd., Tokyo, Japan).

### Detection of inhibitory effect of NAC or cimetidine on drug-induced tissue injury by the fluorescence intensity of the hemolymph

Fifth-instar silkworm larvae on day 1 were fed Silkmate 2S for 1 to 2 days at 27 °C. After the body weight increased to 1.5 to 2.0 g, the silkworms were fasted for 6 h, and 50 μL of 0.4 M NAC or 5 mM cimetidine was injected into the hemolymph from the backside of the larvae using a disposable syringe with a 27G needle. After 30 min, 25 μL of 15% CCl_4_ dissolved in olive oil was injected into the hemocoel using a glass syringe (Hamilton). After incubation at 27 °C for 1 day, the hemolymph was collected for measurement of the fluorescence intensity, as described above.

### Preparation of transparent GFP transgenic silkworms and non-invasive evaluation of drug-induced tissue injury

Fifth-instar larvae on day 1 were fed Silkmate 2S for 1 to 2 days at 27 °C. Then, the larvae were fed Silkmate 2S mixed with 0.5% allopurinol, 0.01% melamine, and 0.5% sodium citrate for 3 days at 27 °C. We confirmed the transparency of the skin of the silkworm larvae and the visibility of the fat body structure from the outside.

Salicylic acid or iron sulfate (50 μl) was injected into the transparent GFP-expressing silkworms. After 16 and 24 h, the exogenous fluorescent image of the silkworm was photographed under excitation light (distance from optical source to silkworm = 35 cm). Mean fluorescence intensity of each silkworm was evaluated by Image J software (ImageJ 1.47t; National Institutes of Health, Bethesda, MD).

After the exogenous fluorescent imaging of the silkworm, several silkworms were dissected to observe the change of fluorescence intensity of fat body and gut. The isolated tissue was observed macroscopically and microscopically under excitation light using a fluorescence microscope (DM4000B; Leica, Wetzlar, Germany).

### Statistical analysis

All experiments were performed at least twice and the data are presented as mean ± standard deviation. The significance of differences between groups was calculated using Student’s *t*-test.

## Additional Information

**How to cite this article**: Inagaki, Y. *et al.* Fluorescence imaging for a noninvasive *in vivo* toxicity-test using a transgenic silkworm expressing green fluorescent protein. *Sci. Rep.* **5**, 11180; doi: 10.1038/srep11180 (2015).

## Supplementary Material

Supplementary Information
