# Supplementary material for: Fluorescence imaging for a noninvasive in vivo toxicity-test using a transgenic silkworm expressing green fluorescent protein
Source: Sci Rep. 2015 Jun 10;5:11180. doi: 10.1038/srep11180 (PMC4462092; doi:10.1038/srep11180)
Supplement: Supplementary Information [file srep11180-s1.pdf]

Supplementary information

Fluorescence imaging for a noninvasive *in vivo* toxicity-test using a transgenic silkworm expressing green fluorescent protein

Yoshinori Inagaki[1], Yasuhiko Matsumoto[1], Masaki Ishii[1], Keiro Uchino[2], Hideki Sezutsu[2], and Kazuhisa Sekimizu[1]

[1]*Laboratory of Microbiology, Graduate School of Pharmaceutical Sciences, The University of Tokyo, 7-3-1 Hongo, Bunkyo-ku, Tokyo 111-0033, Japan.*

[2]*Transgenic Silkworm Research Unit, National Institute of Agrobiological Sciences, 1-2 Owashi, Tsukuba, Ibaraki, 305-8634 Japan*

Correspondence to: Dr. Kazuhisa Sekimizu, Laboratory of Microbiology, Graduate School of Pharmaceutical Sciences, The University of Tokyo, 7-3-1 Hongo, Bunkyo-ku, Tokyo 113-0033, Japan; sekimizu@mol.f.y-tokyo.ac.jp

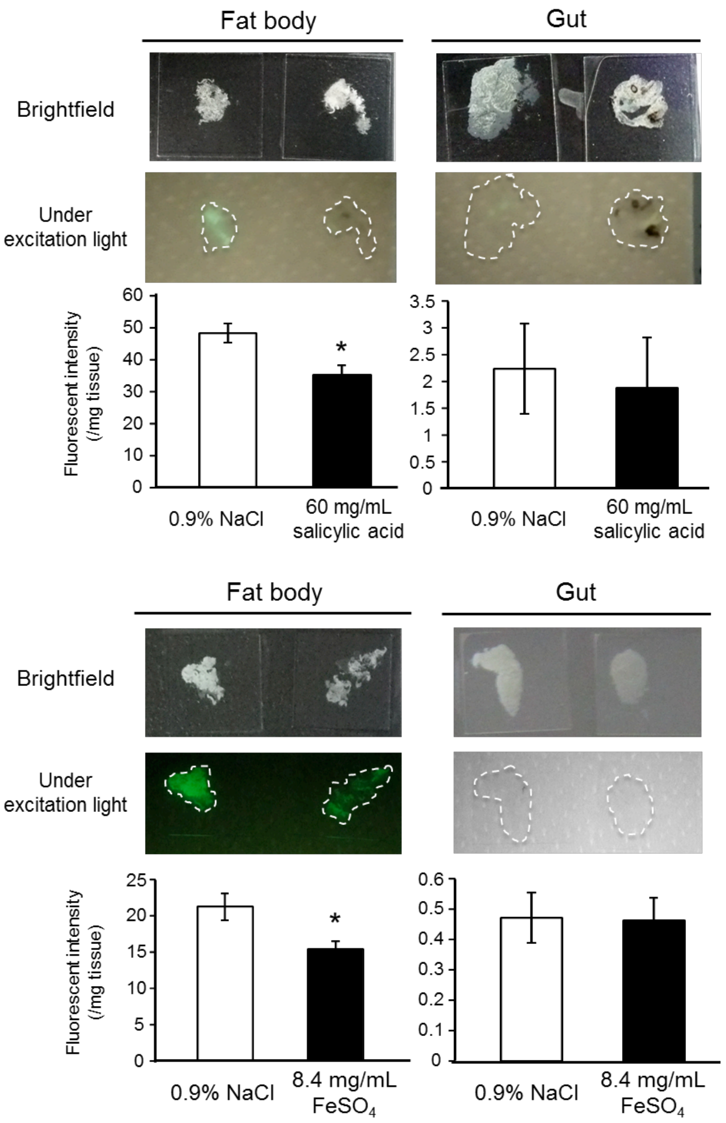

Supplementary figure 1 Effects of salicylic acid and $FeSO_4$ on fluorescence of fat body and gut tissue in transparent GFP transgenic silkworms. Silkworms were made transparent by feeding them a mixed diet (0.5% allopurinol, 0.01% melamine and 0.5% sodium citrate). After fasting for 6 h, silkworms were injected with 0.9% NaCl, 60 mg/mL salicylic acid, or 8.4 mg/mL $FeSO_4$. Fat body and gut were isolated from silkworm 24 h after injection, and fluorescence of each tissue under excitation light was observed. To measure the fluorescent intensity of each tissue, the isolated fat body and gut tissues were homogenized and centrifuged. The fluorescent intensity of each supernatant was measured and compared between the silkworm injected with 0.9% NaCl and 60 mg/mL salicylic acid or 8.4 mg/mL $FeSO_4$. The data shows the fluorescent intensity (mean±SEM) per mg tissue. *$P < 0.05$

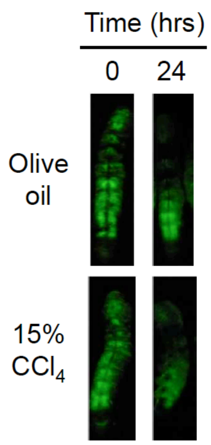

Supplementary figure 2 Effects of olive oil and 15% CCl_4 on whole-body fluorescence of transparent GFP transgenic silkworms. Silkworms were made transparent by feeding them a mixed diet (0.5% allopurinol, 0.01% melamine and 0.5% sodium citrate). After fasting for 6 h, silkworms were injected with olive oil or 15% CCl_4. Whole-body fluorescence of transparent silkworm under excitation light was measured 0 or 24 h after injection.
